# Adenine DNA Glycosylase Activity of 14 Human MutY Homolog (MUTYH) Variant Proteins Found in Patients with Colorectal Polyposis and Cancer

**DOI:** 10.1002/humu.21363

**Published:** 2010-11

**Authors:** Masanori Goto, Kazuya Shinmura, Yusaku Nakabeppu, Hong Tao, Hidetaka Yamada, Toshihiro Tsuneyoshi, Haruhiko Sugimura

**Affiliations:** 1 First Department of Pathology, Hamamatsu University School of MedicineJapan; 2 Division of Neurofunctional Genomics, Department of Immunobiology and Neuroscience, Medical Institute of Bioregulation, Kyushu UniversityJapan; 3 Department of Materials and Life Science, Shizuoka Institute of Science and TechnologyJapan

**Keywords:** base excision repair, 8-hydroxyguanine, MUTYH, MUTYH-associated polyposis, MAP, DNA glycosylase, colorectal cancer

## Abstract

Biallelic inactivating germline mutations in the base excision repair *MUTYH (MYH)* gene have been shown to predispose to MUTYH-associated polyposis (MAP), which is characterized by multiple colorectal adenomas and carcinomas. In this study, we successfully prepared highly homogeneous human MUTYH type 2 recombinant proteins and compared the DNA glycosylase activity of the wild-type protein and fourteen variant-type proteins on adenine mispaired with 8-hydroxyguanine, an oxidized form of guanine. The adenine DNA glycosylase activity of the p.I195V protein, p.G368D protein, p.M255V protein, and p.Y151C protein was 66.9%, 15.2%, 10.7%, and 4.5%, respectively, of that of the wild-type protein, and the glycosylase activity of the p.R154H, p.L360P, p.P377L, p.452delE, p.R69X, and p.Q310X proteins as well as of the p.D208N negative control form was extremely severely impaired. The glycosylase activity of the p.V47E, p.R281C, p.A345V, and p.S487F proteins, on the other hand, was almost the same as that of the wild-type protein. These results should be of great value in accurately diagnosing MAP and in fully understanding the mechanism by which MUTYH repairs DNA in which adenine is mispaired with 8-hydroxyguanine. © 2010 Wiley-Liss, Inc.

## INTRODUCTION

8-Hydroxyguanine (8-OHG) is an oxidized form of guanine (Kasai and Nishimura, 1991), and because 8-OHG can pair with adenine as well as cytosine, formation of 8-OHG in DNA causes a G:C to T:A transversion mutation (Shibutani et al., 1991). MUTYH protein (MIM# 604933), also known as MYH protein, is a DNA glycosylase that catalyzes the removal of adenine mispaired with 8-OHG in double-stranded DNA (Slupska et al., 1999; Shinmura et al., 2000; Tao et al., 2008). Two major MUTYH proteins, i.e., type 1 and type 2, are expressed in human cells as a result of the presence of multiple transcription initiation sites and alternative splicing of mRNA transcripts (Takao et al., 1999; Ohtsubo et al., 2000). Type 1 is composed of 535 amino acids, and because it contains a mitochondrial targeting signal (MTS) in its N-terminal, it is localized in the mitochondria. Type 2 is composed of only 521 amino acid, because it lacks the N-terminal 14 amino acids of type 1, which contain the MTS, and as a result type 2 is localized in the nucleus (Takao et al., 1999; Ohtsubo et al., 2000). The excisional repair activity of the type 2 protein is greater than that of the type 1 protein under certain conditions (Shinmura et al., 2000).

Biallelic inactivating germline mutations in the *MUTYH* gene predispose to MUTYH-associated polyposis (MAP; MIM# 608456), an autosomal recessive disorder characterized by multiple colorectal adenomas and carcinomas (Al-Tassan et al., 2002; Jones et al., 2002; Sampson et al., 2003; Sieber et al., 2003). Since the diagnosis of MAP depends on the level of repair activity of the MUTYH variants encoded in the two *MUTYH* alleles of the patient and the presence of the clinical phenotype characteristic of MAP, even when *MUTYH* gene variations are present in a patient, information on the level of repair activity of the MUTYH variants is indispensable to making the diagnosis of MAP. However, even though more than 80 MUTYH variants have been described in the *MUTYH* gene in colorectal polyposis and colorectal cancer patients (reviewed in Cheadle and Sampson, 2007; Vogt et al., 2009), the effect of only a small number of variations on human MUTYH protein activity has been investigated (Wooden et al., 2004; Bai et al., 2005; Bai et al., 2007; Ali et al., 2008; Kundu et al., 2009; Forsbring et al., 2009; Molatore et al., 2010). One of the reasons for not investigating the effect of more variations is that human MUTYH recombinant proteins cannot be efficiently overexpressed and purified in *Escherichia coli (E. coli)* and baculovirus cultures or in a cell-free system. Thus, even in previous investigations of variant MUTYH proteins, the purified protein fraction also contained multiple other proteins, judging from the photographs of the SDS-PAGE gels. The authors of one paper (Bai et al., 2005) estimated that the purity of the GST-MUTYH fusion proteins used in the analysis was approximately 15%. Too much amount of contamination by other proteins can interfere with accurate determination of the repair activity of variant MUTYH proteins. Thus, improvement of the production and purification system is needed to enable accurate evaluation of the repair activity of variant MUTYH proteins. Moreover, since somatic *APC* (MIM# 611731) mutations occur in the nuclear DNA of a high proportion of MAP tumors (Al-Tassan et al., 2002), it is preferable to evaluate the repair activity of the type 2 protein localized in the nucleus, not the type 1 mitochondrial protein. However, except for the study by Molatole et al. (2010), the repair activity of variants of the type 1 mitochondrial MUTYH form, not the type 2 nuclear form, has been studied in previous studies. Therefore, in the present study we established an experimental system for the purification of MUTYH type 2 recombinant proteins and evaluated 14 type 2 variants, i.e., p.V47E, p.Y151C, p.R154H, p.I195V, p.M255V, p.R281C, p.A345V, p.L360P, p.G368D, p.P377L, p.452delE, p.S487F, p.R69X, and p.Q310X, which correspond to type 1 proteins p.V61E, p.Y165C, p.R168H, p.I201V, p.M269V, p.R295C, p.A359V, p.L374P, p.G382D, p.P391L, p.466delE, p.S501F, p.R83X, and p.Q324X, respectively. All of the above are MUTYH variants that have been identified in patients with colorectal polyposis and/or with colorectal cancer (Halford et al., 2003; Sieber et al., 2003; Aceto et al., 2005; Aretz et al., 2006; Kanter-Smoler et al., 2006; Lejeune et al., 2006; Peterlongo et al., 2006; Russell et al., 2006; Yanaru-Fujisawa et al., 2008). This study assessed the adenine excisional activity of a larger number of MUTYH variants than in previous studies, and the repair activity of the type 2 protein of 11 of the 14 MUTYH variants (p.V47E, p.R154H, p.I195V, p.M255V, p.R281C, p.A345V, p.L360P, p.P377L, p.S487F, p.R69X, and p.Q310X) was examined for the first in this study.

## MATERIALS AND METHODS

### Plasmid construction

The human MUTYH type 2 cDNA sequence was inserted into a pET25b(+) expression vector (Novagen, Darmstadt, Germany). The expression vector for 13 missense-type variants was generated by site-directed mutagenesis with a QuikChange Site-directed Mutagenesis kit (Stratagene, La Jolla, CA). The expression vector for p.R69X and p.Q310X types were constructed by inserting MUTYH cDNA sequence (nucleotides 1-204 and 1-927, respectively) into the pET25b(+) expression vector. All vectors were confirmed by DNA sequencing with a BigDye Terminator Cycle Sequencing Reaction Kit (Applied Biosystems, Tokyo, Japan) and an ABI 3100 Genetic Analyzer (Applied Biosystems).

### Preparation of the recombinant MUTYH proteins

*E. coli* BL21-CodonPlus (DE3)-RP competent cells (Stratagene) were transformed with the MUTYH-pET25b vector and cultured at 37°C until an A_600_ of 0.6. After incubation with 0.1 mM IPTG at 15°C for 12 h, MUTYH-His_6_ protein was purified with TALON metal affinity resins (Clontech, Palo Alto, CA) and a TALON 2-ml disposable gravity column (Clontech). The protein was then dialyzed against buffer containing 10 mM sodium phosphate (pH 7.6), 50 mM NaCl, 0.5 mM DTT, 0.1 mM EDTA, 0.5 mM PMSF, 2 μg/ml pepstatin, 2 μg/ml leupeptin, 50 μM chymostatin, and 10% glycerol. The quality and concentration of MUTYH proteins were determined by using an Agilent 2100 Bioanalyzer (Agilent Technologies, Palo Alto, CA) and Image J software (National Institutes of Health, Bethesda, MD).

### Western blot analysis

Purified recombinant protein was mixed with an equal volume of 2x SDS sample buffer and boiled. A 2 ug protein was subjected to SDS-polyacrylamide gel electrophoresis (PAGE) and electrophoretically transferred to a polyvinylidene difluoride membrane (GE Healthcare Bio-Science Corp., Piscataway, NJ). The membrane was blocked with non-fat milk and incubated with an anti-MUTYH polyclonal antibody (Ohtsubo et al., 2000). After washing, the membrane was incubated with an anti-rabbit HRP-conjugated secondary antibody (GE Healthcare Bio-Science Corp.). The membrane was then washed, and immunoreactivity was visualized with an ECL Plus chemiluminescence system (GE Healthcare Bio-Science Corp.).

### DNA cleavage activity assay

30-mer oligonucleotides containing and not containing a single 8-OHG (5′-CTG GTG GCC TGA C[8-OHG or T]C ATT CCC CAA CTA GTG-3′) were chemically synthesized and purified by PAGE (Japan Bio Services, Saitama, Japan). Complementary oligonucleotides containing an adenine opposite the 8-OHG or T were ^32^P-labeled at the 5′ terminus with a MEGALABEL kit (Takara, Osaka, Japan) and a [γ-^32^P]ATP (PerkinElmer, Tokyo, Japan), and then annealed to oligonucleotides containing a single 8-OHG or T. The reaction mixture containing 20 mM sodium phosphate (pH 7.6), 100 mM NaCl, 0.5 mM DTT, 0.5 mM EDTA, 5 μM ZnCl_2_, 1.5% glycerol, 2.5 nM labeled oligonucleotide, 50 μg/ml BSA, and purified MUTYH protein was incubated at 37°C, and the mixture was treated with 0.1 M NaOH at 95°C for 4 min. After adding denaturing formamide dye to the mixture, it was heated at 95°C for 3 min, and subjected to 20% PAGE. A ^32^P-labeled marker oligonucleotide was used as a size marker for the cleavage products. The radioactivity of intact and cleaved oligonucleotides was quantified by using an FLA-3000 fluoroimage analyzer (Fuji Film, Tokyo, Japan) and ImageGauge software (Fuji Film) (Goto et al., 2009).

### Active site titration and evaluation of the rate constant *k*f

The active site titration and evaluation of the rate constant were performed as described previously (Fersht, 1985; Kundu et al., 2009). A 100 ng amount of total proteins was incubated at 37°C for 0 - 30 min with 5 nM 8-OHG containing substrate and the cleavage products were monitored. To determine the amplitude of the burst (*A*_0_), which is proportional to the active protein fraction concentration, the data were fitted to equation (1):

(1)
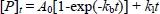


where *[P]_t_* is the cleavage product concentration at time *t* and *k_b_* and *kl* are the rate constants of the burst phase and the linear phase, respectively.

Rate constant, *k_f_*, was evaluated under single-turnover conditions. A 10 nM concentration of active MUTYH enzymes was incubated at 37°C for 0-15 min with 2.5 nM 8-OHG containing substrate and the cleavage products were monitored. To estimate the *k*_f_ the data were fitted to equation (2):

(2)
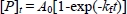


### Mutation nomenclature and reference sequence

Mutation nomenclature is according to den Dunnen and Antonarakis (2000) and den Dunnen and Paalman (2003). The reference sequence for the *MUTYH* gene encoding type 2 protein is accession number NM_001048174.1. The nucleotide numbering system uses the A of the ATG translation initiation start site as nucleotide +1.

## RESULTS

**Figure 1:**
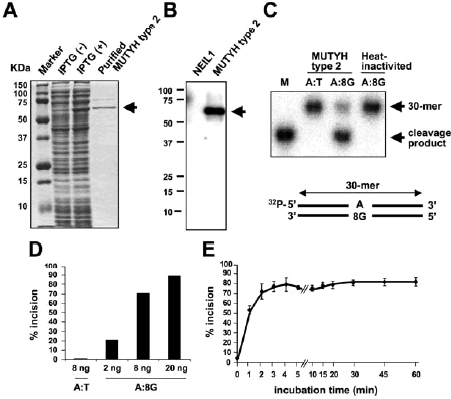
Measurement of the adenine DNA glycosylase activity of wild-type MUTYH type 2 protein. (**A**) Purification of wild-type MUTYH type 2 protein resolved by SDS-polyacrylamide gel electrophoresis (PAGE) and stained with Coomassie Brilliant Blue. Lysates of *E. coli* culture without or with IPTG induction and purified MUTYH type 2 protein are shown. The arrow points to the MUTYH-His_6_ protein band. (**B**) Western blot of purified wild-type MUTYH type 2 protein tagged with His_6_. MUTYH-His_6_ protein is indicated by the arrow. Purified recombinant NEIL1 (MIM# 608844)-His_6_ protein, which was prepared by using pET25b(+) vector (Novagen) and *E. coli* BL21-CodonPlus (DE3)-RP cells (Stratagene) previously (Shinmura et al., 2004), was included as a negative control. (**C**) The DNA glycosylase activity of MUTYH type 2 protein on double-stranded DNA containing an A:8-hydroxyguanine (8-OHG). The MUTYH type 2 protein and a ^32^P-labeled double-stranded oligonucleotides containing or not containing a single 8-OHG mispair were incubated and subjected to 20% PAGE. The intact 30-mer oligonucleotides and cleavage products are indicated by the arrows. Heat-inactivation of the MUTYH protein was accomplished by heating the protein at 100°C for 5 min. 8G means 8-hydroxyguanine. (**D**) Protein concentration dependency of cleavage of DNA containing an A: 8-OHG by MUTYH type 2 protein. The MUTYH protein (2, 8, and 20 ng) was incubated at 37°C for 15 min with a 30-mer oligonucleotide containing an A: 8-OHG or A:T (50 fmole). The amount of cleavage products as a proportion of total oligonucleotides was calculated as % incision. (**E**) Time-course assay of cleavage of DNA containing an A: 8-OHG by MUTYH type 2 protein. The 8 ng amount of MUTYH type 2 protein was incubated at 37°C for 0-60 min with double-stranded oligonucleotide containing an A: 8-OHG (50 fmole). The % incision valves are means ± standard errors of data from three independent experiments.

To overcome the difficulty of preparing highly purified recombinant MUTYH proteins, in this study, we used a pET25b(+) expression vector and BL21-CodonPlus (DE3)-RP *E. coli* host cells for induction of MUTYH expression and a TALON metal affinity resin and gravity column for purification of the MUTYH proteins. Wild-type human MUTYH type 2 protein tagged with His_6_ at its C-terminus was successfully overexpressed in *E. coli* and purified to approximately 90% homogeneity (Figure 1A). The specificity of the purified MUTYH protein was confirmed by Western blotting with anti-MUTYH polyclonal antibody (Figure 1B). Their molecular size of approximately 61 kDa was determined by SDS-PAGE / Coomassie Brilliant Blue (CBB) staining and Western blotting, and it corresponded to their size calculated from the cDNA sequence. The DNA glycosylase activity of the wild-type MUTYH protein was tested by determining its capacity to cleave a double-stranded oligonucleotide containing an adenine mispaired with 8-OHG. The cleavage products were analyzed on a denaturing polyacrylamide gel, and their mobility was compared with that of a marker oligonucleotide. No clear cleavage products were detected when oligonucleotide containing an unmodified A:T base pair was exposed to the MUTYH protein, but cleavage products having the same mobility as the marker oligonucleotide were detected when MUTYH proteins were allowed to react with oligonucleotide containing an A:8-OHG base pair (Figure 1C). No cleavage was detected when allowed to react after heat-inactivation of the MUTYH protein (Figure 1C). The amount of cleavage products was calculated as percent of total oligonucleotides and expressed as % incision, and the % incision of protein substrate containing an A:8-OHG mispair increased in a protein-concentration-dependent manner (Figure 1D). The time-course assay of the cleavage activity of MUTYH protein on substrate containing an A:8-OHG mispair indicated that the amount of cleavage products peaked within 5 min and was almost constant from 5 min to 60 min (Figure 1E). The results showed that highly purified wild-type MUTYH type 2 protein had been obtained with our expression and purification system, and the adenine DNA glycosylase activity of the protein on an A: 8-OHG mispair was satisfactorily detected by our assay. We therefore decided to apply our experimental systems to assessment of the adenine DNA glycosylase activity of various MUTYH variant proteins.

**Figure 2:**
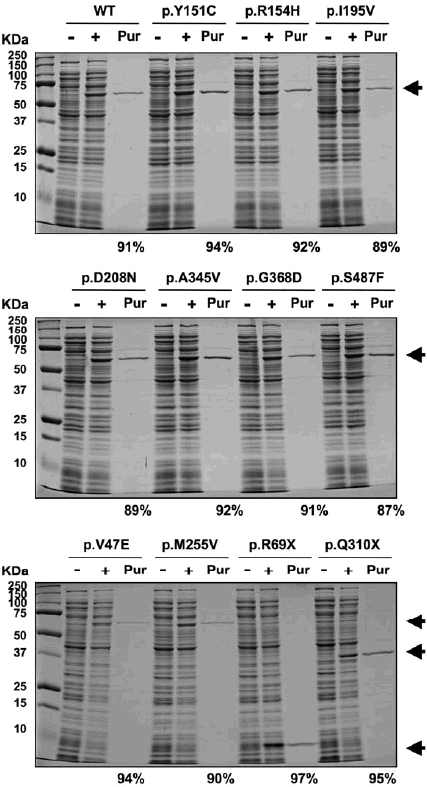
Purification of variant-type MUTYH type 2 recombinant proteins. Each protein was overexpressed and purified under conditions essentially the same as used for the wild-type (WT) protein. Representative results of expression and purification of MUTYH proteins resolved by SDS-PAGE and stained with Coomassie Brilliant Blue are shown. ‘−’ and ‘+’ mean absence and presence, respectively, of IPTG induction, and ‘Pur’ means purified MUTYH type 2 proteins. The arrow points to the MUTYH-His_6_ protein band. The purification level is indicated below the SDS-PAGE panels.

**Figure 3:**
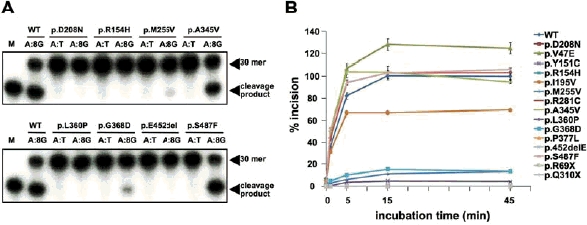
Measurement of the adenine DNA glycosylase activity of variant MUTYH type 2 proteins. (**A**) Representative results of DNA cleavage assays of MUTYH variant proteins are shown. MUTYH proteins (130 fmole) were allowed to act on double-stranded oligonucleotide containing a single A: 8-OHG (8G) mispair at 37°C for 15 min. The reaction mixture was analyzed by 20% PAGE. A ^32^P-labeled marker oligonucleotide was used as a size marker for the cleavage products. The intact 30-mer oligonucleotides and cleavage products are indicated by the arrows. (**B**) Time-course assay of cleavage of DNA containing an A:8-OHG by MUTYH type 2 protein. MUTYH type 2 proteins (130 fmole) were incubated at 37°C for 0 - 45 min with double-stranded oligonucleotide containing an A: 8-OHG (50 fmole). The amount of cleavage products as a proportion of total oligonucleotides was calculated as % incision, and the % incision of each variant-type MUTYH protein is shown relative to that of wild-type (WT) MUTYH protein, which has been set equal to 100. The % incision valves are means ± standard errors of data obtained from three independent experiments in which three independently prepared MUTYH proteins were used.

**Supp. Figure S1:**
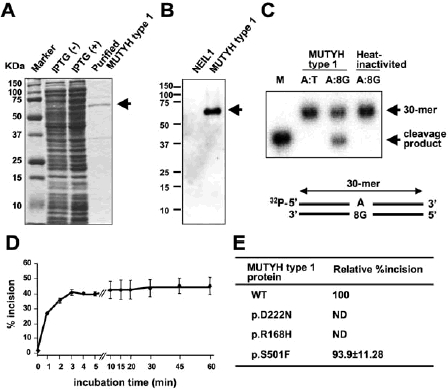
Measurement of DNA glycosylase activity of MUTYH type 1 protein. (**A**) Purification of wild-type MUTYH type 1 protein resolved by SDS-polyacrylamide gel electrophoresis (PAGE) and stained with Coomassie Brilliant Blue (CBB). The human MUTYH type 1 cDNA sequence was inserted into a pET25b(+) expression vector (Novagen, Darmstadt, Germany). *E. coli* BL21-CodonPlus (DE3)-RP competent cells (Stratagene, La Jolla, CA) were transformed with the MUTYH-pET25b vector and cultured at 37°C until an A_600_ of 0.6. After incubation with 0.1 mM IPTG at 20°C for 12 h, MUTYH-His_6_ protein was purified with TALON metal affinity resins (Clontech, Palo Alto, CA) and a TALON 2-ml disposable gravity column (Clontech). The protein was then dialyzed against buffer containing 10 mM sodium phosphate (pH 7.6), 50 mM NaCl, 0.5 mM DTT, 0.1 mM EDTA, 0.5 mM PMSF, 2 μg/ml pepstatin, 2 μg/ml leupeptin, 50 μM chymostatin, and 10% glycerol. Lysates of *E. coli* culture without or with IPTG induction and purified MUTYH type 1 protein are shown. The arrow points to the MUTYH-His_6_ protein band. (**B**) Western blot of purified wild-type MUTYH type 1 protein tagged with His_6_. Purified recombinant NEIL1-His_6_ protein, which was prepared by using pET25b(+) vector (Novagen) and *E. coli* BL21-CodonPlus (DE3)-RP cells (Stratagene) previously (Shinmura et al., 2004), was included as a negative control. Purified recombinant protein was mixed with an equal volume of 2x SDS sample buffer and boiled. A 2 ug protein was subjected to SDS-PAGE and electrophoretically transferred to a polyvinylidene difluoride membrane (GE Healthcare Bio-Science Corp., Piscataway,NJ). The membrane was blocked with nonfat milk and incubated with an anti-MUTYH polyclonal antibody (Ohtsubo et al., 2000). After washing, the membrane was incubated with an anti-rabbit HRP-conjugated secondary antibody (GE Healthcare Bio-Science Corp.). The membrane was then washed, and immunoreactivity was visualized with an ECL Plus chemiluminescence system (GE Healthcare Bio-Science Corp.). MUTYH-His_6_ protein is indicated by the arrow. (**C**) The DNA glycosylase activity of wild-type MUTYH type 1 protein on double-stranded DNA containing an A:8-hydroxyguanine (8-OHG). 30-mer oligonucleotides containing and not containing a single 8-OHG (5′-CTG GTG GCC TGA C[8-OHG or T]C ATT CCC CAA CTA GTG-3′) were chemically synthesized and purified by PAGE (Japan Bio Services, Saitama, Japan). Complementary oligonucleotides containing an adenine opposite the 8-OHG or T were ^32^P-labeled at the 5′ terminus with a MEGALABEL kit (Takara, Osaka, Japan) and a [γ-^32^P]ATP (PerkinElmer, Tokyo, Japan), and then annealed to oligonucleotides containing a single 8-OHG or T. The reaction mixture containing 20 mM sodium phosphate (pH 7.6), 100 mM NaCl, 0.5 mM DTT, 0.5 mM EDTA, 5 μM ZnCl_2_, 1.5% glycerol, 2.5 nM labeled oligonucleotide, 50 μg/ml BSA, and purified MUTYH protein was incubated at 37°C, and the mixture was treated with 0.1 M NaOH at 95°C for 4 min. After adding denaturing formamide dye to the mixture, it was heated at 95°C for 3 min, and subjected to 20% PAGE. A ^32^P-labeled marker oligonucleotide was used as a size marker for the cleavage products. The radioactivity of intact and cleaved oligonucleotides was quantified by using an FLA-3000 fluoroimage analyzer (Fuji Film, Tokyo, Japan) and ImageGauge software (Fuji Film) (Goto et al., 2009). The intact 30-mer oligonucleotides and cleavage products are indicated by the arrows. Heat-inactivation of the MUTYH protein was accomplished by heating the protein at 100°C for 5 min. 8G means 8-hydroxyguanine. (**D**) Time-course assay of cleavage of DNA containing an A:8-OHG by wild-type MUTYH type 1 protein. The MUTYH type 1 protein (260 fmole) was incubated at 37°C for 0 - 60 min with double-stranded oligonucleotide containing an A:8-OHG (50 fmole). The amount of cleavage products as a proportion of total oligonucleotides was calculated as % incision. The % incision values are shown as means ± standard errors of data from three independent experiments. (**E**) DNA glycosylase activities of wild-type MUTYH type 1 protein and their variant proteins on an A:8-OHG substrate. DNA cleavage activities of MUTYH type 1 proteins were measured at 37°C for 15 min. The amount of cleavage products as a proportion of total oligonucleotides was calculated as % incision, and the % incision of each variant-type MUTYH protein is shown relative to that of wild-type (WT) MUTYH protein, which has been set equal to 100. Values are means ± standard errors of data from three independent experiments. ND, not detected.

Fourteen MUTYH variants that had previously been identified in patients with colorectal polyposis and/or colorectal cancer were selected, and their expression vectors were prepared by site-directed mutagenesis. Since the Asp222 in MUTYH type 1 is the active site, and the p.D222N mutant is known not to have DNA glycosylase activity (Wooden et al., 2004), we prepared a type 2-p.D208N construct corresponding to type 1-p.D222N as a negative control. A total of 15 MUTYH type 2 proteins were successfully expressed and purified to a high level of homogeneity (Figure 2). Their molecular sizes determined by SDS-PAGE / CBB staining corresponded with their sizes calculated from their cDNA sequences. Each MUTYH variant protein was almost always reproducibly obtained in the three independent protein preparation (Table 1). Next, we compared the DNA glycosylase activity of each variant protein on oligonucleotide containing an A:8-OHG mispair with that of the wild-type MUTYH type 2 protein (Figure 3 and Table 1). As expected, no clear cleavage products were detected when any of the variant proteins were allowed to act on oligonucleotide containing an unmodified A:T base pair (Figure 3). The adenine DNA glycosylase activity of the MUTYH variant proteins on oligonucleotide containing an A:8-OHG mispair varied (Figure 3 and Table 1). The adenine DNA glycosylase activity of the p.V47E, p.R281C, p.A345V, and p.S487F proteins on the A:8-OHG substrate under conditions of 37°C for 15 min were similar to that of the wild-type protein or only slightly different (102.8% - 128.1%, with activity of the wild-type protein set equal to 100%), whereas p.I195V protein exhibited slightly lower glycosylase activity (66.9%). The p.Y151C protein, p.M255V protein and p.G368D protein exhibited only 4.5%, 10.7%, and 15.2%, respectively, of the glycosylase activity of the wild-type protein, and the adenine DNA glycosylase activity of the p.R154H, p.L360P, p.P377L, p.452delE, p.R69X, and p.Q310X proteins as well as of the p.D208N negative control protein was almost at the background level. We also attempted to determine whether the recombinant type 1 variant proteins had a level of repair activity that was similar to that of the corresponding type 2 proteins. We randomly chose type 1-p.R168H and type 1-p.S501F, which correspond to type 2-p.R154H and type 2-p.S487F, respectively, and found that the activity level of the type 1 and type 2 proteins of at least these two MUTYH variants in comparison with the wild-type protein is similar (Supp. Figure S1A-E). We also estimated the rate constant *k*_f_ of some type 2 proteins on the A:8-OHG substrate after correction for the active enzyme fraction as described previously (Fersht, 1985; Kundu et al., 2009). The rate constant for adenine excision by wild-type protein was 0.524, and similar to the constant for p.R281C (*k*_f_ = 0.489) (Table 2). However, the *k*_f_ value of p.M255V was 0.024 and more than 20-fold lower than that of the wild-type protein, indicating that the glycosylase activity of p.M255V was severely reduced. The above findings indicate that the adenine DNA glycosylase activity of nine of the 14 MUTYH type 2 variant proteins tested is severely impaired.

**Table 1:** DNA glycosylase activity of 14 variants of MUTYH type 2 protein on DNA containing adenine mispaired with 8-hydroxyguanine

MUTYH type 2 protein	Type of mutation^a^	Purified protein yield (ng) per 10 ml culture^b^	Relative % incision^c^
WT		4365, 3562, 3454	100
p.D208N	negative control	2073, 1826, 1808	extremely severely defective
p.V47E	c.140T>A, missense	1018, 1893, 1916	128.1±5.20
p.Y151C	c.452A>G, missense	5485, 4779, 2835	4.5±0.21
p.R154H	c.461G>A, missense	894, 1501, 2109	extremely severely defective
p.I195V	c.583A>G, missense	1733, 1102, 671	66.9±0.35
p.M255V	c.763A>G, missense	2446, 1726, 2488	10.7±0.47
p.R281C	c.841C>T, missense	912, 934, 447	103.0±1.39
p.A345V	c.1034C>T, missense	2564, 2435, 3864	103.5±5.43
p.L360P	c.1079T>C, missense	152, 303, 120	extremely severely defective
p.G368D	c.1103G>A, missense	2486, 1246, 2270	15.2±0.71
p.P377L	c.1130C>T, missense	885, 626, 430	extremely severely defective
p.452delE	c.1353_1355delGGA, inframe deletion	822, 614, 364	extremely severely defective
p.S487F	c.1460C>T, missense	1099, 984, 706	102.8±2.31
p.R69X	c.205C>T, nonsense	6289, 5245, 6289	extremely severely defective
p.Q310X	c.928C>T, nonsense	918, 5726, 2771	extremely severely defective

a The reference sequence for the *MUTYH* gene encoding type 2 protein is accession number NM_001048174.1.

b Amount of MUTYH proteins purified from 10 ml of *E. coli* culture expressing MUTYH type 2 protein. Each protein was purified three times and has been listed in this table.

c The DNA cleavage activity of MUTYH protein was measured under conditions of 37°C for 15 min. The amount of cleavage products as a proportion of total oligonucleotides was calculated as % incision, and the % incision of each variant-type MUTYH protein is shown relative to that of wild-type (WT) MUTYH protein, which has been set equal to 100. Values are means ± standard errors of data obtained from three independent experiments in which three independently prepared MUTYH proteins were used.

**Table 2:** Active yield and rate constant kf evaluated for MUTYH type 2 protein on DNA containing adenine mispaired with 8-hydroxyguanine

MUTYH type 2 protein	Active protein yield (μg) per 1L culture^a^	*k*_f_(min^−1^)^b^
WT	2706, 1248,2045	0.524±0.034
p.M255V	58,72,75	0.024±0.004
p.R281C	944, 1128,751	0.489±0.044

a Values were obtained by incubating 100 ng of total proteins with 5 nM of substrate. Results obtained with three separate protein preparations are shown.

b Values were obtained by incubating 10 nM of active MUTYH enzyme and 2.5 nM of substrate. Values are means ± standard errors of data obtained from three independent experiments using independently prepared proteins.

## DISCUSSION

The cumulative results of recent screenings of colorectal polyposis patients for *MUTYH* mutations have revealed many *MUTYH* gene variants (reviewed in Cheadle and Sampson, 2007; Vogt et al., 2009), but the repair activity of the type 2 protein of most of the variants has never been tested. In this study we improved the method of expressing and purifying the recombinant MUTYH type 2 proteins and assessed the adenine DNA glycosylase activity of various type 2 proteins. The results revealed that the p.V47E, p.R281C, p.A345V, and p.S487F proteins largely retain adenine removing activity, but that the adenine removing activity of the p.I195V protein is mildly impaired and the activity of the p.Y151C, p.R154H, p.M255V, p.L360P, p.G368D, p.P377L, p.452delE, p.R69X, and p.Q310X proteins is severely impaired. This information should be of great help in accurately diagnosing MAP and managing MAP patients.

The adenine DNA glycosylase activity of the type 1 or type 2 MUTYH variants p.Y151C, p.G368D, p.P377L, p.452delE, and p.S487F has been assessed previously (Wooden et al., 2004; Ali et al., 2008; Kundu et al., 2009; Forsbring et al., 2009; Molatore et al., 2010). The results showed that the repair activity of p.Y151C, p.G368D, p.P377L, and p.452delE was impaired while that of p.S487F was retained, findings that were consistent with our own, and the consistency confirms the reliability of the results of our study. Interestingly, a slight difference (approximately 5% vs. approximately 15%) in repair activity was observed between p.Y151C and p.G368D in our study, and the same difference was reported in several previous papers. Nielsen et al. (2009) recently reported finding that MAP patients homozygous for a p.G368D allele have a milder clinical phenotype than MAP patients homozygous for a p.Y151C allele. The difference in adenine removing activity between the p.Y151C protein and p.G368D protein may be related to the difference in clinical phenotype.

Retention of DNA glycosylase activity by the human MUTYH proteins p.V47E, p.R281C, and p.A345V as well as mild impairment of the activity of p.I195V and severe impairment of the activity of p.R154H, p.M255V, p.L360P, p.R69X, and p.Q310X were documented for the first time in this study. Although no analyses of crystal structure of the MUTYH has been reported, based on the cumulative results of previous studies of the biochemistry of MUTYH (reviewed in Cheadle and Sampson, 2007; Ali et al., 2008), p.R154, p.M255, and p.L360 are located in regions suspected of being important to catalytic activity or substrate recognition. Moreover, the p.R154, p.M255, and p.L360 in MUTYH protein are conserved among *E. coli, Mus musculus, Rattus norvegicus, Pan troglodytes, Canis familiaris*, and *Homo sapiens*. Substitution of any of these amino acids appears to result in a functional abnormality, resulting in severe reduction of adenine DNA glycosylase activity on DNA containing an A:8-OHG mispair. The loss of large parts of the MUTYH protein in the frameshift-type variant proteins p.R69X and p.Q310X may be responsible for the severe impairment of their adenine removing activity. An amino acid substitution in p.V47E, p.R281C, p.A345V, and p.S487F did not greatly affect their adenine DNA glycosylase activity. These results are consistent with the prediction of a possible impact of an amino acid substitution on the structure and function of MUTYH type 2 protein by the PolyPhen-2 program (http://genetics.bwh.harvard.edu/pph2/index.shtml) (Adzhubei et al., 2010). Since p.V47E, p.R281C, p.A345V, and p.S487F are not located in the region suspected of being important to catalytic activity or in a well-conserved position of the substrate recognition region, the amino acid localization may be one of the reasons for the retention of enzymatic activity. In the future, a crystal structure of the MUTYH protein alone and covalently complexed with DNA, in conjunction with the present findings on MUTYH variants, should contribute to establishing further correlations between the structure and repair function of the MUTYH protein.

Highly homogeneous MUTYH type 2 recombinant protein was prepared by using the method described in this study. As far as we have been able to determine in a review of the literature, the level of purification of the proteins in our study was higher than level of purification estimated from the SDS-PAGE images of MUTYH type 1 or type 2 proteins in previous papers and purification levels described in the other papers. Use of *E. coli* BL21-CodonPlus (DE3)-RP cells, which contain extra copies of the genes that encode the tRNAs of rare *E. coli* codons, is thought to be responsible for the increase in level of MUTYH protein expression, because the *MUTYH* gene contains many rare codons. The expression conditions (temperature and IPTG concentration), combination of expression vector and competent cells, and metal-affinity purification conditions are also thought to possibly have contributed to the improvement in the level of purification in this study. Since genetic screening of colorectal polyposis patients for *MUTYH* mutations will continue to be performed worldwide, the same as genetic screening for *APC* mutations (reviewed in Lynch et al., 2008), our method of expression and purification of human MUTYH protein should be useful for assessing MUTYH variants newly identified by genetic screening as well as MUTYH variants that have not been examined.

As shown in Supp. Figure S1E, the activity level of the type 1 and type 2 proteins of two MUTYH variants in comparison with the wild-type protein was similar. Although comparisons were not made for the other variants, it may not always be necessarily to study the type 2 form when analyzing MUTYH protein. However, since type 2, and not type 1, is a nuclear form (Takao et al., 1999; Ohtsubo et al., 2000), and somatic *APC* mutations occur in the nuclear DNA of MAP tumors (Al-Tassan et al., 2002), we think that evaluation of type 2 variants is likely to be more preferable when we investigate the possible pathogenic role of MUTYH in MAP. Assessment of the glycosylase activity level of the wild-type type 1 and type 2 proteins showed that the activity of type 2 was greater than that of type 1 (Figure 1 and Supp. Figure S1), a finding that is consistent with a previous report (Shinmura et al., 2000). The reason for the difference in the catalysis of adenine excision between the type 1 and type 2 proteins is unclear. One point that requires caution is that there is evidence that the type 1 protein is processed during mitochondrial transport in cells and the mature form of the protein never been identified, and thus the repair activity of the full-length type 1 may not necessarily reflect the activity *in vivo*. With regard to the method of evaluating MUTYH variant proteins, since MUTYH possesses suppressive activity against G:C to T:A mutations caused by 8-OHG (Yamane et al., 2003) and biallelic *MUTYH* inactivation leads to somatic *APC* mutation in MAP tumors (Al-Tassan et al., 2002), analysis of the mutation rate of the *APC* gene in MUTYH variant-expressing cells may be an alternative way of evaluating the level of repair activity of a MUTYH variant. A combination of mutation rate analysis and DNA glycosylase analysis would provide more definitive proof of the pathogenicity of a MUTYH variant.
